# Deuterated docosahexaenoic acid protects against oxidative stress and geographic atrophy‐like retinal degeneration in a mouse model with iron overload

**DOI:** 10.1111/acel.13579

**Published:** 2022-03-08

**Authors:** Yingrui Liu, Brent A. Bell, Ying Song, Kevin Zhang, Brandon Anderson, Paul H. Axelsen, Whitney Bohannan, Martin‐Paul Agbaga, Hui Gyu Park, Genevieve James, J. Thomas Brenna, Karsten Schmidt, Joshua L. Dunaief, Mikhail S. Shchepinov

**Affiliations:** ^1^ F.M. Kirby Center for Molecular Ophthalmology Scheie Eye Institute Perelman School of Medicine at the University of Pennsylvania Philadelphia Pennsylvania USA; ^2^ Department of Pharmacology Perelman School of Medicine at the University of Pennsylvania Philadelphia Pennsylvania USA; ^3^ Departments of Cell Biology and Ophthalmology University of Oklahoma Health Sciences Center and the Dean McGee Eye Institute Oklahoma City Oklahoma USA; ^4^ Dell Pediatric Research Institute University of Texas at Austin Austin Texas USA; ^5^ Retrotope, Inc. Los Altos California USA

**Keywords:** age‐related macular degeneration, deuterium, docosahexaenoic acid, iron, isotope effect, lipid peroxidation, oxidative stress, polyunsaturated fatty acid

## Abstract

Oxidative stress plays a central role in age‐related macular degeneration (AMD). Iron, a potent generator of hydroxyl radicals through the Fenton reaction, has been implicated in AMD. One easily oxidized molecule is docosahexaenoic acid (DHA), the most abundant polyunsaturated fatty acid in photoreceptor membranes. Oxidation of DHA produces toxic oxidation products including carboxyethylpyrrole (CEP) adducts, which are increased in the retinas of AMD patients. In this study, we hypothesized that deuterium substitution on the *bis*‐allylic sites of DHA in photoreceptor membranes could prevent iron‐induced retinal degeneration by inhibiting oxidative stress and lipid peroxidation. Mice were fed with either DHA deuterated at the oxidation‐prone positions (D‐DHA) or control natural DHA and then given an intravitreal injection of iron or control saline. Orally administered D‐DHA caused a dose‐dependent increase in D‐DHA levels in the neural retina and retinal pigment epithelium (RPE) as measured by mass spectrometry. At 1 week after iron injection, D‐DHA provided nearly complete protection against iron‐induced retinal autofluorescence and retinal degeneration, as determined by *in vivo* imaging, electroretinography, and histology. Iron injection resulted in carboxyethylpyrrole conjugate immunoreactivity in photoreceptors and RPE in mice fed with natural DHA but not D‐DHA. Quantitative PCR results were consistent with iron‐induced oxidative stress, inflammation, and retinal cell death in mice fed with natural DHA but not D‐DHA. Taken together, our findings suggest that DHA oxidation is central to the pathogenesis of iron‐induced retinal degeneration. They also provide preclinical evidence that dosing with D‐DHA could be a viable therapeutic strategy for retinal diseases involving oxidative stress.

## Graphical abstract

DHA oxidation is central to the pathogenesis of iron‐induced retinal degeneration. Deuterium substitution on the *bis*‐allylic sites of DHA in photoreceptor membranes could prevent iron‐induced retinal degeneration by inhibiting oxidative stress and lipid peroxidation.

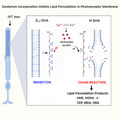

**Abbreviations**
4‐HHE: 4‐hydroxyhexenal
AF: autofluorescence
AMD: age‐related macular degeneration
BAF: blue autofluorescence
CEP: carboxyethylpyrrole
cSLO: confocal scanning laser ophthalmoscopy
D‐DHA: deuterated docosahexaenoic acid
DHA: docosahexaenoic acid
DPA: docosapentaenoic acid
DTA: docosatetraenoic acid
EP: ethylpyrrole
HOHA: 4‐hydroxy‐7‐oxo‐5‐heptenoic acid
HNE: 4‐hydroxynonenal
IHC: immunohistochemistry
IRAF: infrared autofluorescence
IVT: intravitreal
L‐Ft: ferritin light chain
LPO: lipid peroxidation
MDA: malondialdehyde
OBA: oxobutanoic acid ester
OCT: optical coherence tomography
ONL: outer nuclear layer
PUFAs: polyunsaturated fatty acids
ROS: reactive oxygen species
RPE: retinal pigment epithelium
SEM: standard error of the mean

## 1 INTRODUCTION

Oxidative stress plays a major role in the pathogenesis of neurodegenerative and retinal diseases (Shichiri, 2014). The retina is subject to oxidative damage because of free radicals generated by abundant mitochondria, especially when photosensitizers in the mitochondria are exposed to blue light (King et al., 2004). Bisretinoids produced as byproducts of the visual cycle are also blue light photosensitizers (Sparrow et al., 2000). Polyunsaturated fatty acids (PUFAs), which are abundant in plasma and mitochondrial membranes, are especially vulnerable to oxidative stress, since reactive oxygen species (ROS) initiate a lipid peroxidation (LPO) chain reaction.

**FIGURE 1:**
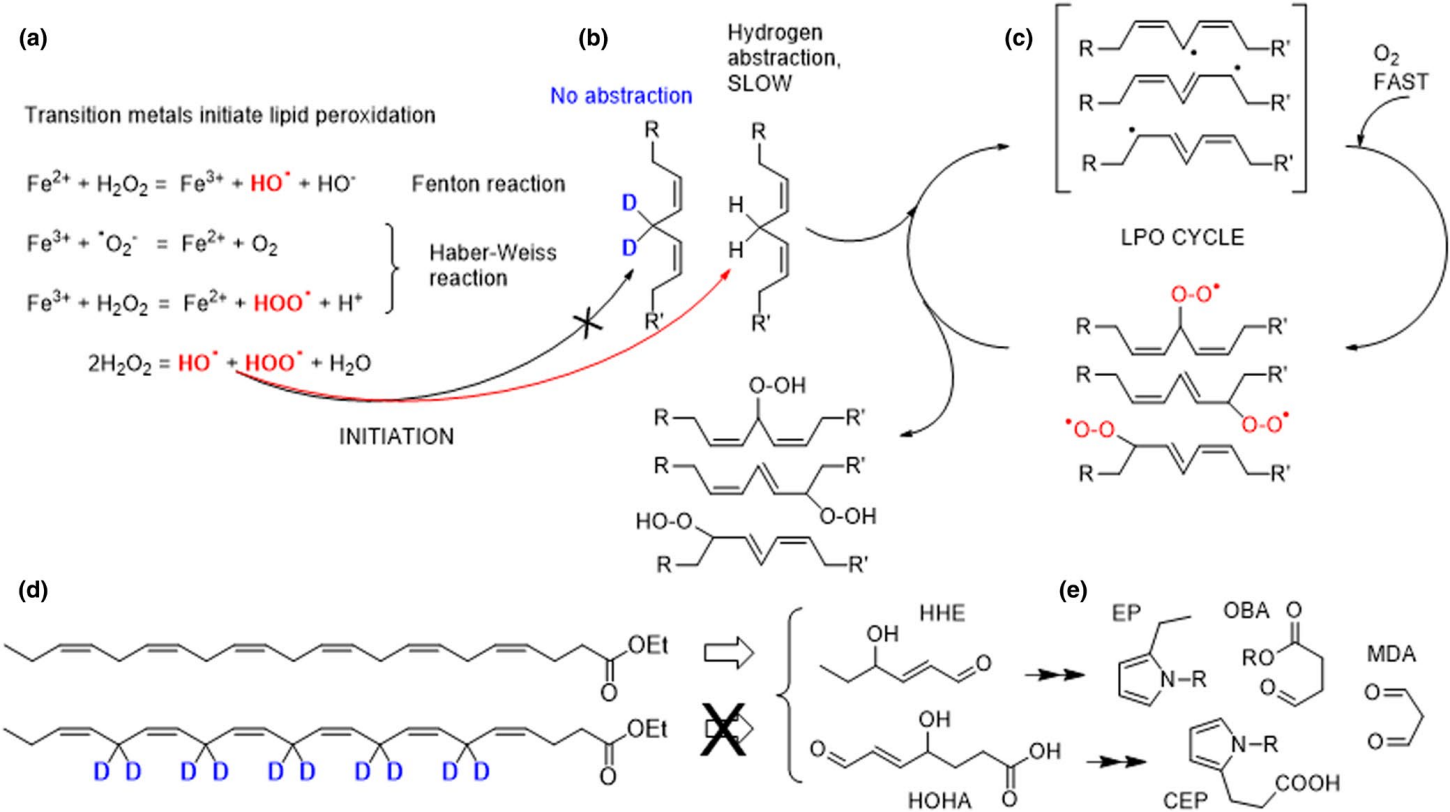
Chemical diagram of the steps in iron‐catalyzed lipid peroxidation of phospholipids containing DHA and the formation of CEP. Fe catalyzes hydroxyl radical generation through the Fenton reaction and Haber‐Weiss reaction (a). ROS‐driven hydrogen abstraction off bis‐allylic sites generates free radicals, which rapidly react with oxygen to form lipid peroxyl radicals (b). These newly formed ROS species then abstract bis‐allylic hydrogen atoms from the neighboring PUFAs, thus sustaining the LPO chain reaction cycle (c). D‐DHA was used in this study (d). DHA peroxidation generates multiple oxidation products including reactive carbonyls such as HHE and HOHA, which can give rise to protein modifications, including OBA, CEP, and MDA adducts (e). The substitution of deuterium for hydrogen atoms inhibits the rate‐limiting step of ROS‐driven abstraction off *bis*‐allylic sites

The abstraction of *bis*‐allylic hydrogens is the rate‐limiting step of ROS‐driven PUFA oxidation. Substitution of deuterium atoms for hydrogen atoms at *bis*‐allylic sites can slow down the LPO chain reaction due to an isotope effect (Shchepinov, 2020) (Figure 1). PUFAs cannot be synthesized *de novo* from carbon sources, for example, acetate. Typically, linoleic acid and alpha‐linolenic acid, respectively, serve in the diet as the major precursors for biosynthesis of all the n‐6 and n‐3 PUFAs (Kothapalli et al., 2020). This ensures that D‐PUFAs are incorporated into mitochondrial and cellular membranes after oral dosing, replacing a fraction of the PUFAs naturally occurring in membranes, and conferring resistance to oxidative stress and LPO. D‐PUFAs have been studied in multiple conditions involving oxidative stress and LPO (Andreyev et al., 2015; Berbée et al., 2017; Hill et al., 2011, 2012). A deuterated version of linoleic acid (11,11‐D_2_‐Lin, RT001) inhibited LPO and rescued cell death in both animal models and clinical trials in several neurodegenerative diseases, including Friedreich's ataxia (FRDA) (Zesiewicz et al., 2018), infantile neuroaxonal dystrophy (INAD) (Kinghorn et al., 2015), and progressive supranuclear palsy (PSP) (Angelova et al., 2021). D‐PUFAs also reduced LPO and hold therapeutic potential in preclinical studies for Alzheimer's (Raefsky et al., 2018), Parkinson's (Beal et al., 2020; Shchepinov et al., 2011), and Huntington's diseases (Hatami et al., 2018).

Oxidative stress has been implicated in several retinal diseases, including age‐related macular degeneration (AMD) (Beatty et al., 2000), light‐induced damage (Cheng et al., 2019; Tanito et al., 2005), iron‐related retinal degeneration (Katz et al., 1993; Shu et al., 2020), Leber's hereditary optic neuropathy (Kirches, 2011), and retinitis pigmentosa (Campochiaro et al., 2015; Komeima et al., 2006; Tuson et al., 2009). Docosahexaenoic acid (cervonic acid; DHA, C22:6, *n*‐3) is the most abundant PUFA in the retina, representing up to 40% of all total fatty acids in human rod photoreceptor outer segments (Fliesler & Anderson, 1983). DHA is crucial for the integrity of photoreceptors and visual function (Benolken et al., 1973). While ingestion of DHA‐rich fatty fish is associated with lower AMD risk, *n*‐3 PUFA supplementation has shown no appreciable benefits in patients with AMD (Souied et al., 2013) or retinitis pigmentosa (Hoffman et al., 2014). Moreover, high doses of DHA may increase risk in conditions involving oxidative stress, due to its high sensitivity to oxidation (Tanito & Anderson, 2009). The addition of DHA to the human RPE cell line ARPE‐19 (Dunn et al., 1996) increased oxidative stress and LPO under high‐intensity light exposure (Liu et al., 2014). Levels of carboxyethylpyrrole (CEP), a protein adduct specifically derived from the oxidation of DHA, are elevated in retinal tissues (Beatty et al., 2000) and plasma (Ardeljan et al., 2011; Ni et al., 2009) from patients with AMD. Furthermore, immunization of mice with CEP adducts led to an AMD‐like retinal degeneration (Hollyfield et al., 2008). These pieces of evidence suggest that nonenzymatic oxidation of DHA in the retina may play a crucial role in the pathogeneses of retinal disorders involving oxidative stress.

In this study, we investigate the impact of deuterated DHA against oxidative stress and LPO in mice with iron‐induced oxidative stress in the retina. We previously reported a mouse model given intravitreal (IVT) injection of iron and found increased oxidative stress and CEP in the retina, followed by retinal pathologies similar to human AMD, including geographic atrophy of the RPE (Liu et al., 2021). Here, we fed mice a diet containing an envelope of D‐DHA isotopologues, with most prevalent being 6,6,9,9,12,12,15,15,18,18‐D10‐(4Z, 7Z, 10Z, 13Z, 16Z, 19Z)‐docosa 4,7,10,13,16,19‐hexaenoic acid ethyl ester for 11 weeks followed by a washout in a pharmacokinetic study to establish a dosing regimen for efficient retinal uptake. To determine the protective effect of D‐DHA against LPO, we fed mice a diet containing D‐DHA for 1–4 weeks before giving an IVT injection of iron or control saline. In mice fed with D‐DHA for 4 weeks, >50% substitution of DHA with D‐DHA in the neural retina was observed. This regimen provided nearly complete protection against iron‐induced retinal damage by inhibiting oxidative stress and DHA oxidation.

## 2 RESULTS

### 2.1 Dietary D‐DHA efficiently incorporated into the neural retina and RPE cells

**FIGURE 2:**
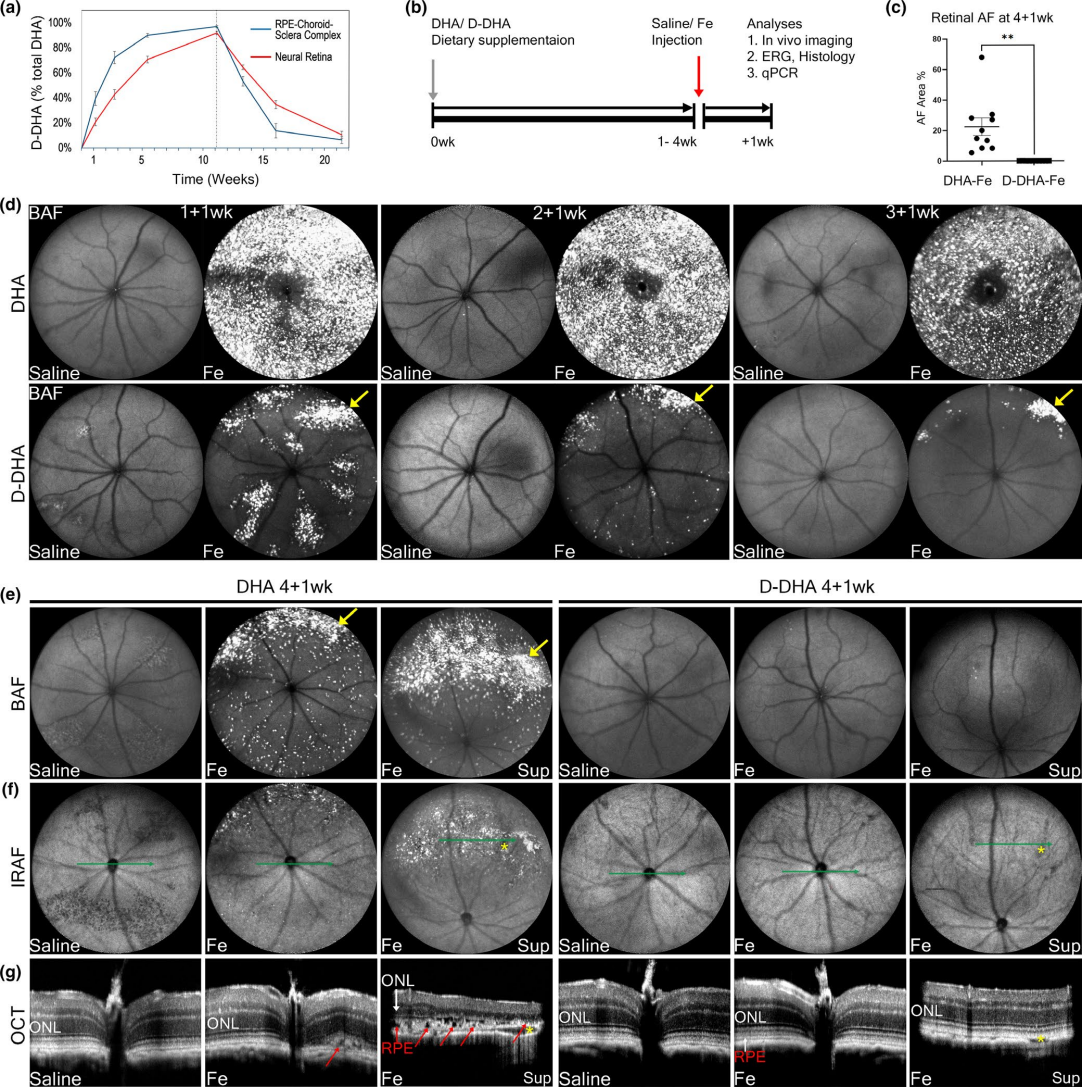
D‐DHA protected against iron‐induced retinal autofluorescence and degeneration. Mice were fed with D‐DHA for 77 days, followed by a switch to DHA for 74 days for a total of 151 days of feeding. Graph shows %D‐DHA in neural retina and RPE‐choroid (a). Timeline shows that mice were fed with either D‐DHA or DHA for 1 week, 2 weeks, or 4 weeks beginning at 2 months age, then given intravitreal injection of iron in one eye and control normal saline in the other. Mice were continued on their respective diets until their final evaluation (b). cSLO and OCT imaging were performed at 1 week after IVT iron versus saline injection. Graph shows retinal AF area in BAF cSLO images from mice fed with 4 weeks of DHA or D‐DHA at 1 week after iron injection (designated 4+1 wk) (c). Representative BAF cSLO images in mice fed D‐DHA or DHA for 1 week, given IVT injections, then euthanized a week later (1+1 wk), or fed D‐DHA for two weeks, given IVT injections, then euthanized a week later (2+1 wk), etc. (d and e), IRAF cSLO images (f), and horizontal OCT b scans (g) are shown. SLO, scanning laser ophthalmoscopy; OCT, optical coherence tomography; BAF, blue autofluorescence; IRAF, infrared autofluorescence; ONL, outer nuclear layer. Yellow arrows indicate hyper‐AF spots induced by iron; red arrows indicate RPE degeneration. Green lines indicate the position and orientation of horizontal OCT b scans in panel e, yellow stars indicate the vortex vein that was used as a landmark for the corresponding position of the OCT scan in IRAF SLO images. N=10 mice/group in c; Error bars indicate mean ± SEM. (**p < 0.01)

To determine the pharmacokinetics of ocular D‐DHA uptake, incorporation, and elimination from the neural retina and RPE/choroid/sclera, twelve‐week‐old C57BL/6J mice were fed a 0.5% D‐DHA containing diet (0.5 g D‐DHA/100g food, Table S1) for 77 days followed by an additional 74‐day washout phase with DHA. At 4 weeks, >55% of the DHA in the retina was D‐DHA rising to >60% at 5 weeks (Figure 2a). At similar time points, D‐DHA in the RPE/choroid/sclera was >80%. Washout in the RPE/choroid/sclera was similarly more rapid than retina. Uptake and elimination followed classic first‐order kinetics. Based on the accretion and elimination data, two‐month‐old mice were fed with diets containing either D‐DHA or natural DHA control for 1, 2, 3, and 4 weeks before the IVT injection of iron and saline control (Figure 2b). In order to better approximate typical DHA doses in human prescription omega‐3 supplements (e.g., 1,500 mg/day DHA in Lovaza), the D‐DHA and DHA experimental mouse diets were adjusted to 0.25% instead of 0.5% for most of the study. On a 0.25% D‐DHA diet, retinal D‐DHA levels exceeded 50% at 5 weeks (52.2 ± 1.5% and 55% of total DHA by our GC‐ and LC‐based MS methods, respectively), regardless of whether eyes were injected with iron or control saline (Table 1).

**TABLE 1:** D‐DHA content as a percentage of D‐DHA +DHA in neural retina and RPE from mice fed with D‐DHA or DHA at 4 weeks, given IVT injections, and then continued on the D‐DHA diet for another week

Treatment	Tissue	% D substitution
D‐DHA + Fe	RPE cells	59.3 ± 3.9
D‐DHA + saline	RPE cells	60.8 ± 2.1
D‐DHA + Fe	neural retina	55.0 ± 3.3
D‐DHA + saline	neural retina	55.0 ± 1.8

### 2.2 D‐DHA protected against iron‐induced retinal autofluorescence (AF) and degeneration

We previously reported IVT iron‐induced retinal AF and degeneration (Liu et al., 2021). To evaluate the protective effect of 0.25% D‐DHA diet, confocal scanning laser ophthalmoscopy (cSLO) and optical coherence tomography (OCT) were employed for *in vivo* imaging at 1 week after injections. For the cSLO imaging, both blue autofluorescence (BAF) to detect bisretinoids and near‐infrared autofluorescence (IRAF) to detect melanin in the RPE and choroid were performed. Fundus AF in BAF images from mice at 1 week after iron injection was quantified by ImageJ Software (Figure 2c). Images of mice fed with natural DHA showed significantly higher levels of BAF than of those fed D‐DHA. Those fed with natural DHA displayed intense hyper‐autofluorescent spots, representing photoreceptor layer undulations, as well as autofluorescent RPE and myeloid cells (Liu et al., 2021) (Figure 2d,e). These same retinas imaged with IRAF showed hyper and hypo autofluorescence in the superior retina. Since IRAF detects melanin, these changes most likely correlate with the RPE atrophy and anterior migration observed by histology (see below). BAF and IRAF images of mice fed with D‐DHA revealed a dose‐dependent reduction of iron‐induced retinal AF in mice fed with D‐DHA for 1, 2, 3, and 4 weeks before the iron injection (Figure 2d,e; Figure S2). Optical coherence tomography (OCT) scans of mice fed with natural DHA showed marked thinning of the outer nuclear layer, a discontinuous or absent ellipsoid zone in the superior retina, and RPE degeneration at 1 week after iron injection (Figure 2g). In contrast, mice fed with D‐DHA for 4 weeks showed complete protection of retinal structure in OCT scans (Figure 2g).

The extent to which D‐DHA replaced natural DHA was determined by LC/MS in pellets of the control diet containing 0.25% natural DHA, the experimental diet containing 0.25% D‐DHA, and microdissected samples of neural retina and RPE from mice fed the experimental diet for 4 weeks, given IVT iron or control saline, and then continued on the experimental diet for another week. We confirmed that 22.6% of DHA in the control diet consisted of isotopomers due to natural abundance of carbon‐13 (Raefsky et al., 2018) and no D‐DHA. Therefore, signals from the 327.2/283.2 transition represent 78.4% of the total DHA. The experimental diet contained no detectable natural DHA, but rather a distribution of deuterium‐substituted DHA isotopologues (Figure S1), which is a consequence of the D‐DHA preparation method (Smarun et al., 2017; Wang et al., 2021). After corrections were applied for natural abundance of carbon‐13 (Raefsky et al., 2018), we determined that the D_10_‐DHA isotopologue comprised 45.6% of the D‐DHA species, with D_8_‐DHA, D_9_‐DHA, D_11_‐DHA, and D_12_‐DHA isotopologues comprising the balance. Therefore, signals from the 337.2/293.2 transition represent 45.6% of the D‐DHA. With these corrections applied, D‐DHA isotopologues as a percentage of total DHA (i.e., D‐DHA +DHA) were determined to be 59.3–60.8% in isolated RPE, and 55.0% in neural retina. There were no significant differences between iron‐treated and saline‐control eyes (Table 1).

### 2.3 D‐DHA protected retinal function and histologic structure

**FIGURE 3:**
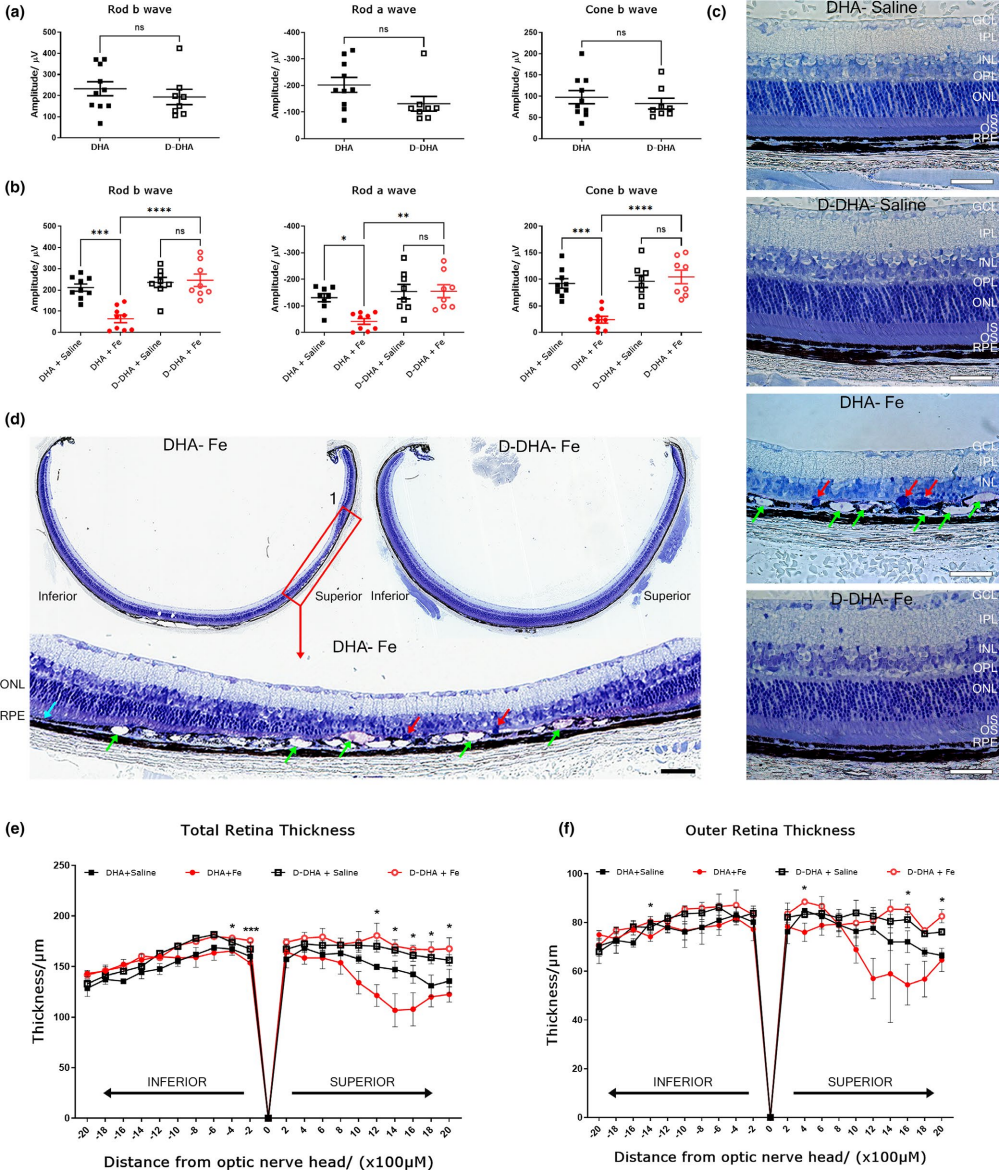
D‐DHA protected retinal function and structure against iron injection. Graphs showing electroretinography amplitudes 4 weeks after dietary dosing of either D‐DHA or DHA (a), then re‐conducted at 1 week after an intravitreal injection of iron or saline (b). Toluidine blue staining was conducted on plastic sections prepared at 1 week after injections (c and d). Enlarged image (red box1) is from section from mouse fed with DHA diet for 4 weeks then given IVT iron and euthanized a week later. Spider graphs show the mean thickness of each retinal layer. Error bars indicate mean ± SEM of total retinal thicknesses and outer retina thickness (ONL to RPE) in the ventral (inferior) – dorsal (superior) axis at the positions indicated on the x‐axis (e and f). Green arrows indicate RPE degeneration. Red arrows indicate cells that have infiltrated the region between the ONL and RPE; these may be myeloid or migrating RPE cells. Two‐sample t‐tests were performed to compare the total retinal thickness and outer retinal thickness between DHA‐Fe group and D‐DHA‐Fe group at each different location. All statistical comparisons were made using SAS v9.4 (SAS Institute Inc., Cary, NC). No correction for multiple comparisons was performed due to the exploratory nature of this small study. Error bars indicate mean ± SEM. *p < 0.05. Scale bar: 50 μM. N = 8‐10/group for electroretinography. N=3/group for retina thickness measures

Electroretinography was conducted on mice fed with D‐DHA or natural DHA for 4 weeks to evaluate retinal function. In mice not given IVT injections, dosing with D‐DHA caused no significant difference in the rod‐b wave, rod‐a wave, and cone‐b wave amplitudes compared to those fed with natural DHA. Thus, using this measure, incorporation of D‐DHA had no impact on retinal function (Figure 3a). In mice fed with control DHA, 1 week after iron injection, the rod b‐wave, rod a‐wave, and cone‐b wave amplitudes were significantly decreased in the eyes injected with iron compared with saline control, consistent with iron‐induced retinal damage. Iron injected eyes from mice with ≥50% retinal D‐DHA had marked protection of rod b‐wave, rod a‐wave, and cone‐b wave amplitudes compared to the DHA plus iron injection group (Figure 3b). There was no significant difference between the saline‐injected eyes and the iron‐injected eyes from mice on the D‐DHA diet, indicating complete functional protection.

Toluidine blue staining was performed on plastic sections to examine retinal histology. At 1 week after iron injection, the outer nuclear layer (ONL) and photoreceptor inner/outer segments were absent overlying RPE degeneration and atrophy (Figure 3c,d). Mice fed with D‐DHA showed complete protection of retinal structure against the toxicity of iron injection (Figure 3c,d). Quantification of total retina thickness and outer retina thickness in IVT iron injected eyes from DHA fed mice displayed a reduction in the superior retina. In contrast, IVT iron injected eyes from D‐DHA‐fed mice were significantly protected and not different from saline‐injected eyes (Figure 3e,f). Taken together, ≥50% retinal D‐DHA substitution led to complete protection of retinal function and structure against iron‐induced oxidative damage.

### 2.4 D‐DHA prevented the formation of CEP, a unique oxidation product of DHA

**FIGURE 4:**
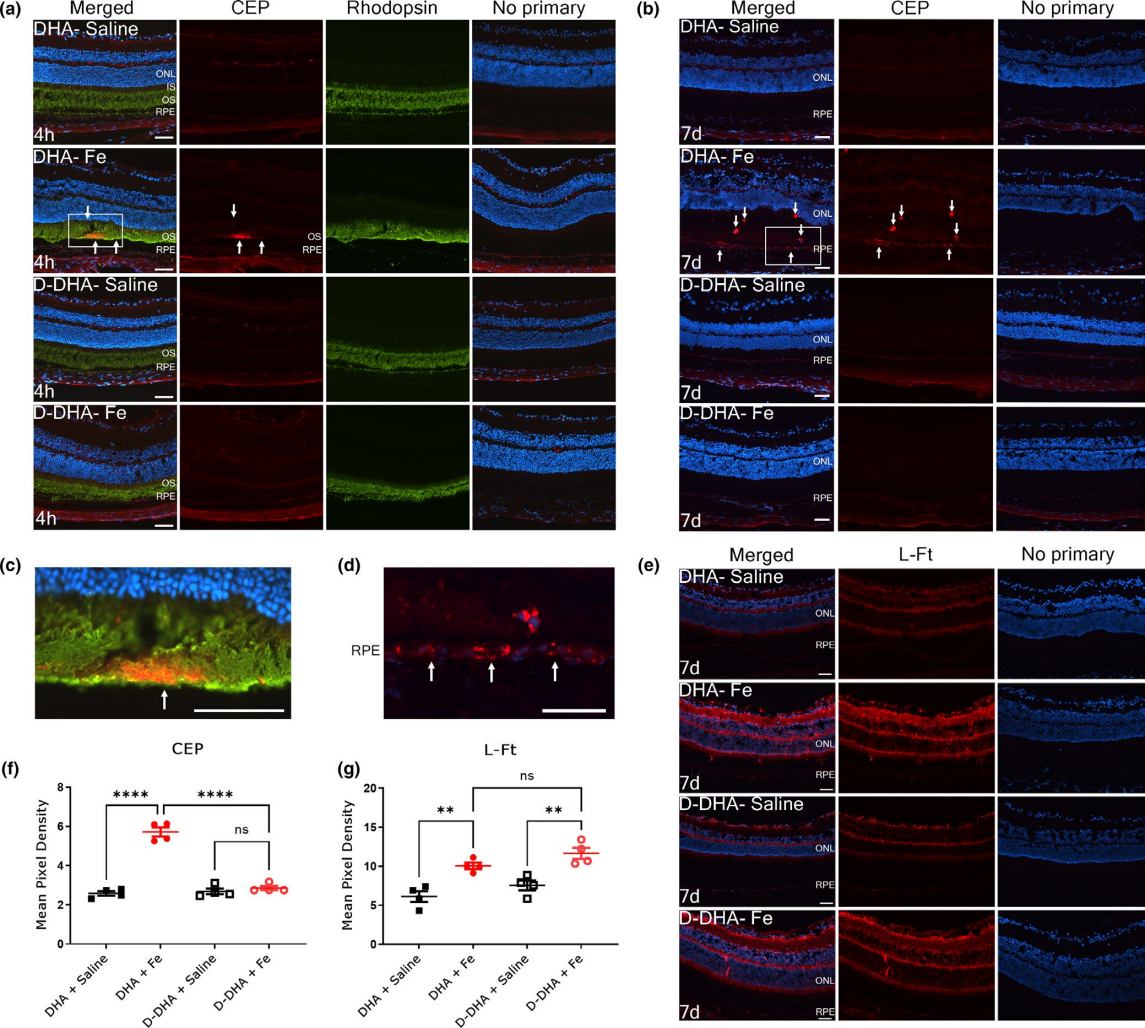
D‐DHA prevented the formation of CEP, an oxidized product specifically derived from DHA oxidation. Epifluorescence photomicrographs of co‐labeling for carboxyethylpyrrole (CEP‐red) and rhodopsin (green) on cryosections from mice fed with 4 weeks of D‐DHA or DHA at 4 hr after intravitreal injection of iron or saline (a). Immunolabeling for CEP at 1 week after injections (b). Enlarged image of co‐labeling for CEP and rhodopsin in a (c). Enlarged image of immunolabeling for CEP in b (d). Immunolabeling for L‐Ft at 1 week after injections (e). Quantification of pixel density of immunolabeling for CEP (f) and L‐Ft (g). White arrows indicate immunolabeling for CEP. CEP, carboxyethyl pyrrole; INL, inner nuclear layer; ONL, outer nuclear layer; RPE, retinal pigmented epithelium. Representative images are shown from N=4 mice/group. Scale bar: 50 µm. Error bars indicate mean ± SEM. **p < 0.01 and ****p < 0.0001

Iron‐catalyzed peroxidation of phospholipids containing DHA leads to unique carboxyethylpyrrole (CEP) adducts not formed from any other PUFA. CEP has been detected by IHC in human AMD eyes and mouse retinas, including those from mice receiving IVT iron (Crabb et al., 2002; Ebrahem et al., 2006; Liu et al., 2021). To test whether D‐DHA could protect against iron‐induced CEP formation, mice were fed with D‐DHA or DHA for 4 weeks prior to IVT injection of iron or control saline. Cryosections were prepared at 4 hr and 1 week after injections. Co‐labeling for CEP and rhodopsin was conducted to assess and localize CEP. At 4 hr after injection, increased immunolabeling for CEP was present in rhodopsin co‐labeled photoreceptor outer segments in IVT iron injected eyes of DHA fed mice but not in IVT iron injected eyes of D‐DHA‐fed mice (Figure 4a). At 1 week after injection, immunolabeling for CEP localized to RPE and infiltrating myeloid cells in IVT iron injected eyes of DHA fed mice, which may come from phagocytosed oxidized photoreceptor outer segments (Figure 4b). Immunolabeling for CEP was undetected in D‐DHA fed mice. These results indicate that iron induced the accumulation of CEP, and D‐DHA at ≥50% retinal substitution prevented the accumulation of CEP by inhibiting DHA oxidation.

Immunolabeling for ferritin light chain (L‐Ft) was conducted to assess retinal iron levels and localization, since L‐Ft protein levels are increased in response to elevated intracellular iron (Song et al., 2014). At 1 week after saline injection, L‐Ft weakly labeled the ganglion cell layer, outer plexiform layer, and inner segment layers (Figure 4e). Increased L‐Ft staining was observed in the inner plexiform layer, outer plexiform layer, and inner segments in both the DHA/IVT iron and D‐DHA/IVT iron mice (Figure 4e). These two groups were not different from each other, indicating that D‐DHA did not prevent IVT iron‐induced iron accumulation in retinal cells; instead, D‐DHA blocked its downstream toxic effects. Quantification of pixel density for CEP and L‐Ft label was conducted using ImageJ software (Figure 4f,g), and quantitatively verified the results described above.

### 2.5 D‐DHA protected against mRNA changes indicative of iron‐induced retinal cell death, oxidative stress, and inflammation

**FIGURE 5:**
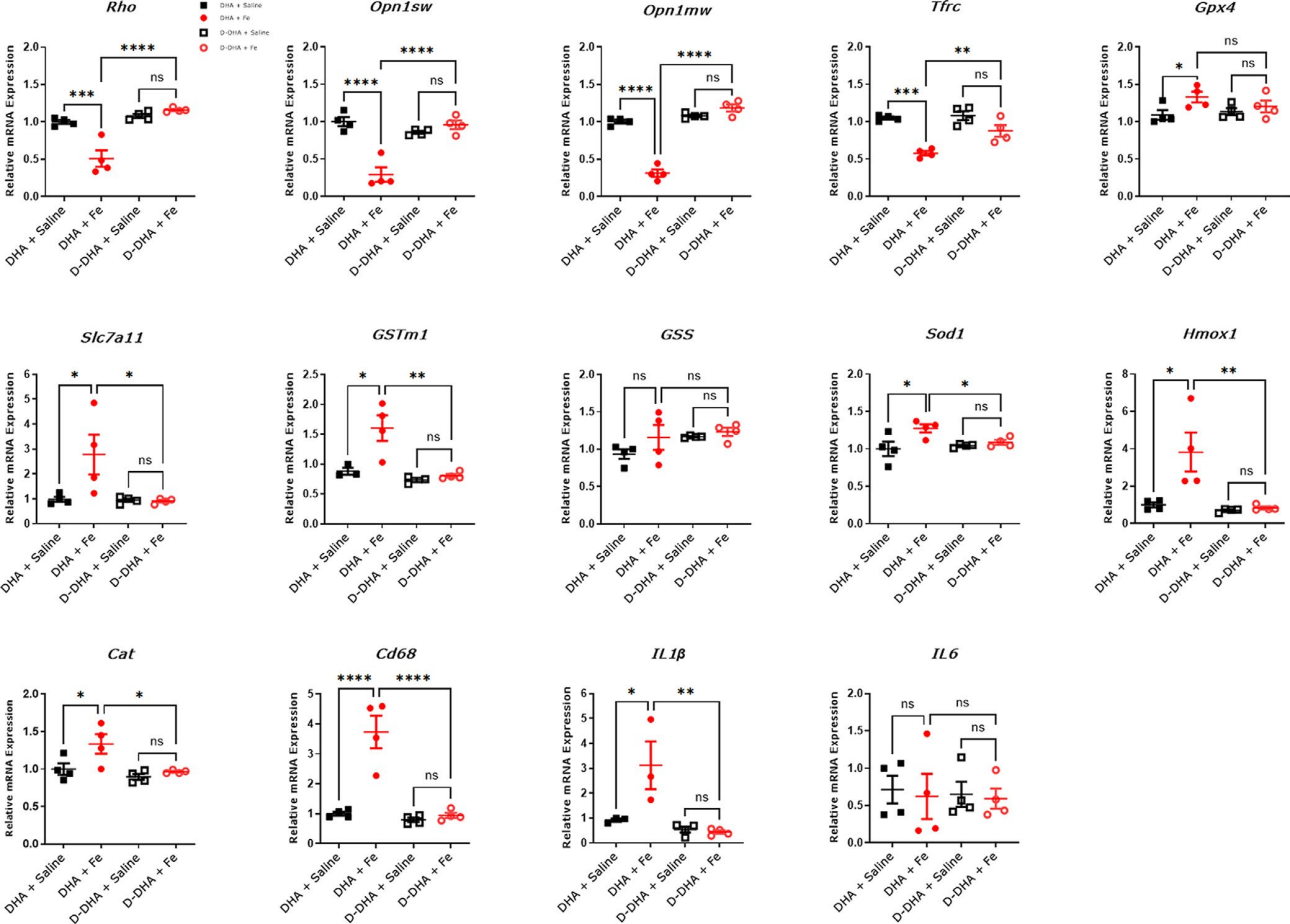
qPCR indicates that D‐DHA protected against iron‐induced oxidative stress, inflammation, and retinal cell death. Relative mRNA levels in the neural retina of the indicated genes from mice fed with 4 weeks of D‐DHA or DHA at 1 week after iron or saline injection. Error bars indicate mean ± SEM. N=3–4 mice/group. (*p < 0.05, **p < 0.01, ****p < 0.0001, and ****p < 0.0001)

Quantitative PCR was used to evaluate mRNA changes in the neural retinas of mice fed with D‐DHA or DHA for 4 weeks. Cell type‐specific, iron regulating, antioxidant, and inflammation‐related genes were evaluated at 1 week after iron or saline injections. The mRNA levels of the rod‐specific gene *rhodopsin* (*Rho*), cone‐specific gene *cone opsin1 medium wave sensitive*, and *short wave sensitive* (*Opn1mw and Opn1sw*) were measured to assess the stress and differentiation of rod and cone photoreceptors. The mRNA levels of *Rho*, *Opn1sw*, and *Opn1mw* were significantly decreased in the neural retinas of mice fed with DHA that received IVT iron, compared to IVT saline controls. In contrast, there was no change in these mRNAs in the neural retinas of D‐DHA/IVT iron mice relative to IVT saline controls (Figure 5). The mRNA levels of transferrin receptor (*Tfrc*) which are inversely related to intracellular iron levels, can be used as an indicator of intracellular iron levels (Song et al., 2016). At 1 week after iron injection, *Tfrc* mRNA levels in the neural retina were significantly decreased in DHA/IVT iron and D‐DHA/IVT iron mice indicating iron loading in the neural retinas of both groups. These two groups had slightly different *Tfrc* levels, perhaps as a result of loss of some photoreceptors in the DHA/IVT iron group (Figure 4). The mRNA levels of antioxidants *solute carrier family 7 member 11 (SLC7A11)*, *glutathione peroxidase 4 (GPX4)*, *glutathione S*‐*transferase isoform m1(GSTm1)*, *glutathione synthesis (GSS)*, *catalase (Cat)*, *heme oxygenase 1 (Hmox1)*, and *superoxide dismutase 1 (Sod1)* were measured to investigate oxidative stress. The mRNA levels of *Slc7a11*, *Gpx4*, *GSTm1*, *Cat*, *Hmox1*, and *Sod1* were significantly increased in the neural retinas of DHA/IVT iron compared with saline‐injected eyes. This upregulation of antioxidants was prevented in D‐DHA/IVT iron eyes, with no significant difference between iron and saline‐injected eyes in mice fed with D‐DHA. The mRNA levels of *IL1β*, *IL6*, and *cluster of differentiation 68* (*Cd68*) were detected to investigate the retinal inflammation. The mRNA levels of *IL1β* and *Cd68* were significantly increased in DHA/IVT iron retinas compared to saline controls but were not increased in the D‐DHA/IVT iron retinas. The mRNA levels of *Glutathione*‐*synthase (GSS)* and *IL6* were not increased by IVT iron in mice on either diet (Figure 5). Taken together, ≥50% retinal D‐DHA can significantly protect against iron‐induced oxidative stress, photoreceptor cell damage, and inflammation in the neural retina.

### 2.6 D‐DHA prevented iron‐induced geographic atrophy development

**FIGURE 6:**
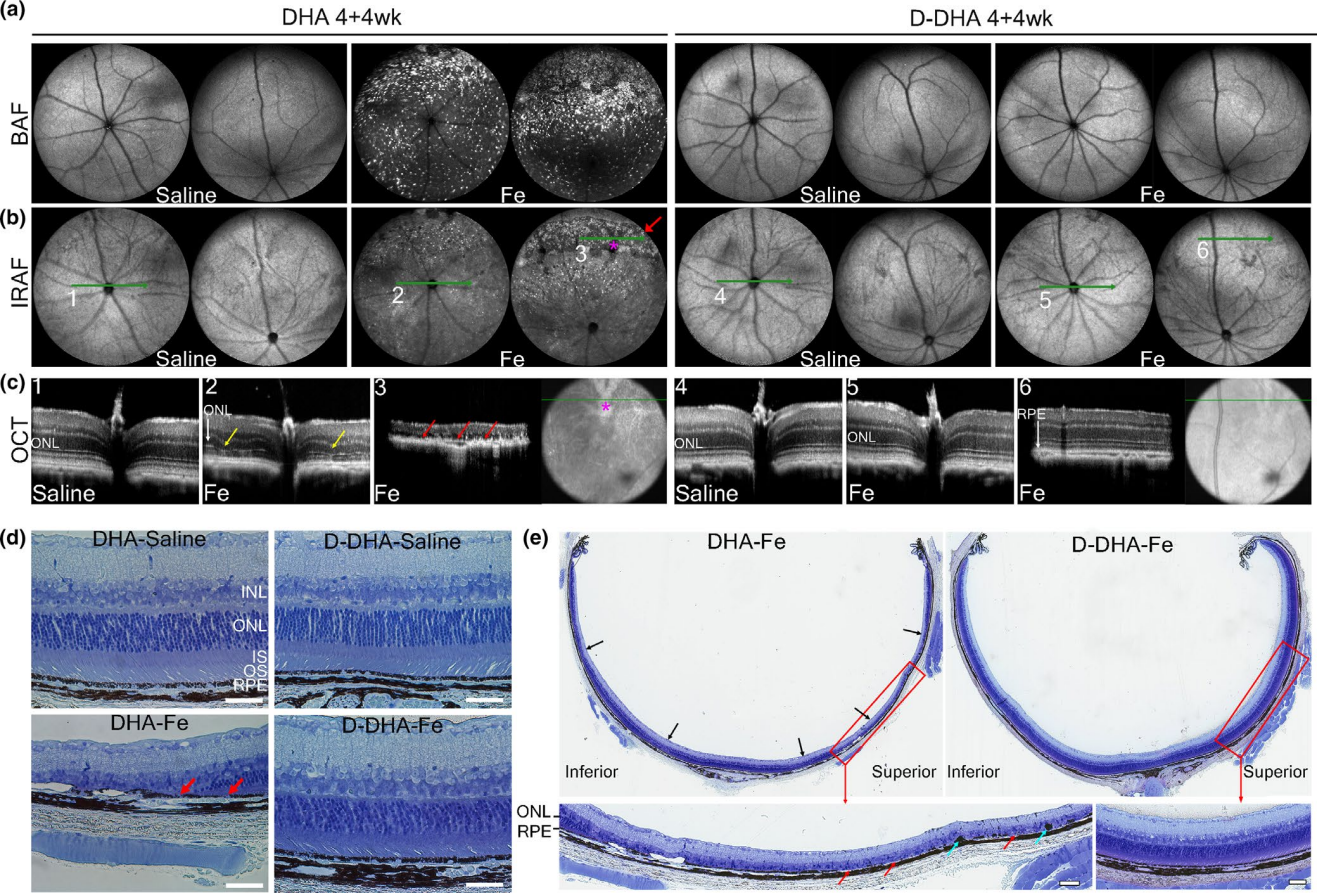
D‐DHA prevented iron‐induced acute RPE atrophy and progressive geographic atrophy development. Mice were fed with 4 weeks of D‐DHA or DHA before receiving intravitreal injection of iron in one eye and control normal saline in the other. cSLO and OCT images were acquired at 4 weeks after iron or saline injection. Representative BAF cSLO images (a), IRAF cSLO images (b), and OCT scans (c) are shown. Toluidine blue staining was conducted on plastic sections prepared at 4 weeks after injections (d and e). Green lines indicate the positions of horizontal OCT scans. Red arrow indicates region of hyper‐AF and hypo‐AF lesions in IRAF cSLO image, corresponding to representative atrophic RPEs in OCT scans. Yellow arrows indicate ONL thinning in OCT scans. Red arrows indicate atrophic RPE in OCT scan. Blue arrows indicate pigmented cells between the ONL and RPE layers. These may be myeloid or migrated RPE cells. Representative images are shown from N=4 mice/group. Scale bar: 50 µm

Mice given D‐DHA for 4 weeks showed complete retinal protection 1 week after iron injection (Figure 2). Our previous study showed geographic atrophy in the superior retina within a month of IVT iron injection (Liu et al., 2021). To evaluate whether D‐DHA could protect against geographic atrophy in this model, mice were continued on their respective diets for 4 weeks after iron or saline injection. At this time point, BAF and IRAF images displayed hypo‐AF in the superior retinas of mice fed with DHA, similar to geography atrophy (Figure 6a,b). This corresponded to representative photoreceptor (yellow arrows) and RPE degeneration (red arrows) in OCT scans (Figure 6c). The absence of outer retinal layers indicates photoreceptor degeneration throughout the OCT scan (Figure 6c, #3). The posterior hypertransmission into the choroid indicates RPE atrophy. D‐DHA is fully protected against the geographic atrophy development (Figure 6a–c). cSLO and OCT scans were obtained from multiple mice, and all displayed the protective effect of D‐DHA on chronic retinal degeneration (Figure S3). Taken together, ≥50% retinal D‐DHA provided long‐term protection against the geographic atrophy development induced by iron.

## 3 DISCUSSION

Herein, we tested the hypothesis that inhibition of DHA oxidation might prevent oxidative stress and retinal degeneration in a mouse model with retinal iron overload. We previously reported IVT iron‐induced retinal AF, oxidative stress, accumulation of carboxyethylpyrrole (CEP), a DHA‐specific oxidation product, and photoreceptor degeneration followed by progressive geographic atrophy, replicating features of human AMD (Liu et al., 2021). In this study, we have found that dosing of D‐DHA completely protected against all these iron‐induced retinal changes.

We found that mice fed with D‐DHA for 1, 2, and 3 weeks prior to the iron injection showed a dose‐dependent reduction in iron‐induced retinal AF and retinal degeneration with >50% protective effects already observed at >30% retinal D‐DHA substitution levels (Figure S4). After D‐DHA reached 50% retinal substitution levels in mice fed with D‐DHA for 4 weeks prior to the iron injection, we observed complete protection of retinal structure and function.

D‐DHA inhibited oxidative stress and LPO, which is particularly pernicious because of its autocatalytic radical chain reaction cycle and nonenzymatic nature. Hydrogen abstraction at the *bis*‐allylic sites is the rate‐limiting step of LPO. PUFAs deuterated at the *bis*‐allylic positions inhibit this step due to the isotope effect (King et al., 2004). D‐DHA prevented oxidative stress‐induced increases in mRNA levels of antioxidants *GSTm1*, *Catalase*, *Sod1*, *Hmox1*, *Gpx4*, and *Slc7a11*. In addition, immunolabeling of CEP was undetected in the retina of mice with ≥50% retinal D‐DHA substitution levels prior to iron injection, suggesting the deuteration can inhibit the oxidation of DHA and the accumulation of its toxic derivative CEP, contributing to retinal protection. CEP (Figure 1e) is a DHA‐specific, adduct‐forming oxidation product (Organisciak et al., 2012). CEP adducts have been found increased in drusen deposits (Crabb et al., 2002) and plasma (Gu et al., 2003, 2009) of AMD patients, and elevated in the retinas of rodents after intense light exposure (Collier et al., 2011; Organisciak et al., 2012). Mice immunized with CEP adducts accumulated complement component‐3 in Bruch's membrane, drusen deposits underneath the RPE, and RPE degeneration, features of dry AMD (Hollyfield et al., 2008). CEP adducts also stimulated neovascularization *in vivo* through a VEGF‐independent pathway (Ebrahem et al., 2006).

LPO is detrimental to cells in multiple ways. It makes lipid bilayers leaky and stiff. On a chemical level, LPO generates small molecule species such as lipid hydroperoxides, prostaglandin‐like isoprostanes, and isoketals which have primarily detrimental effects (Halliwell & Gutteridge, 2015). Another group of LPO products implicated in numerous pathologies comprises activated carbonyls, including malondialdehyde, 4‐HNE (from n‐6 PUFA), and 4‐HHE (from n‐3 PUFA). These are highly reactive (Figure 1e) and can irreversibly cross‐link phospholipids, proteins, and cause DNA transversions. By virtue of inhibiting LPO, D‐PUFAs reduce the levels of these compounds (Kinghorn et al., 2015; Kothapalli et al., 2020; Moriguchi et al., 2001; Raefsky et al., 2018; Shchepinov et al., 2011). Moreover, D‐PUFAs can cross‐protect various PUFAs; a membrane‐incorporated D‐PUFA protects other PUFAs in this membrane by terminating the LPO chain reaction (Shchepinov, 2020). For example, the presence of the n‐6 PUFA D2‐linoleic acid in lipid bilayers down‐regulated not just 4‐HNE but also 4‐HHE formation (Raefsky et al., 2018).

Our previous study showed a threshold protective effect of various D‐PUFAs on the stability of liposomes under oxidative stress, revealing a strong protective effect of D‐DHA, which efficiently inhibited the LPO even when present at 1‐2% fraction of the total PUFAs in a lipid membrane in vitro (Firsov et al., 2019). Herein, mice with ≥50% D‐DHA incorporation in the neural retina and RPE cells showed a complete protection effect against the oxidative damage induced by iron. This is probably because of the high content of DHA in the retina and up to 30 mol% of rod outer segment membrane phospholipids carrying twin DHA acyl chains (supraenoic phospholipids with more than six double bonds) (Aveldaño & Bazán, 1983). This unique feature of photoreceptor outer segments requires ≥50% D‐DHA levels to completely insulate proximal unprotected supraenoic DHA chains from each other. The high levels of hydroxyl radicals generated by Fe through the Fenton reaction and the subsequent LPO cycle in this drastic model demand high concentrations of retinal D‐DHA while lower levels might be sufficient in less severe oxidative conditions.

D‐DHA inhibited the oxidative damage‐associated inflammation in the neural retina. At 1 week after injection, the mRNA levels of *IL1β* and *Cd68* were significantly increased in the iron injected neural retina from mice fed with DHA but showed no significant difference between iron and saline‐injected neural retinas from mice fed with D‐DHA. Oxidative stress and lipid peroxides can induce the inflammatory response, including the infiltration and activation of microglia and macrophages, and the secretion of pro‐inflammatory cytokines such as *IL1*‐*β*, *IL*‐*6*, *TNF*‐*α*, and others. Overall, our results suggest that D‐DHA can prevent the neuroinflammation induced by iron.

D‐DHA showed long‐term protection against geographic atrophy. Mice with ≥50% retinal D‐DHA levels were completely protected against the chronic development of geographic atrophy in the superior retina, compared to mice fed with DHA. Moreover, we found that IVT iron‐induced damage appears to be less severe with the DHA diet than our previous study in mice fed with “regular chow” (LabDiet 5001) (Liu et al., 2021). We previously observed iron‐induced retinal AF throughout the retina in mice fed with LabDiet 5001 at 1 week after iron injection, and “kidney bean” shaped geographic atrophy always occurred at 4 weeks after iron injection (Liu et al., 2021). In the current study, we observed that iron‐induced AF was more limited to the superior retina in mice fed with DHA for 4 weeks at 1 week after iron injection (Figure S2), and the “kidney bean” shaped geographic atrophy only occurred in some, but not all mice (Figure S3), which was correlated with the amount of superior region AF one week after iron injection. Additional investigation will be required to determine the basis of the more severe iron‐induced retinal degeneration in mice on LabDiet 5001, which differs significantly from the DHA control diet used in this study. Since the only difference between the DHA diet and D‐DHA diet used herein is whether DHA is deuterated, our results suggest that D‐DHA leads to long‐term protection against photoreceptor and RPE degeneration induced by iron overload.

D‐PUFAs have been reported to inhibit LPO in several mouse models of neurological diseases associated with oxidative stress, including Parkinson's disease (Angelova et al., 2015; Shchepinov et al., 2011), Alzheimer's disease (Elharram et al., 2017; Raefsky et al., 2018), Huntington's disease (Hatami et al., 2018), and infantile neuroaxonal dystrophy (Kinghorn et al., 2015). RT001 (D2‐Lin ethyl ester) has been tested in clinical trials of Friedreich's ataxia (Zesiewicz et al., 2018) and infantile neuroaxonal dystrophy (Kinghorn et al., 2015) showing notable safety and tolerability. Here, for the first time, we report the protective effect of D‐DHA in retinal disease *in vivo*, using a mouse model replicating features of human AMD. Our results indicate that D‐DHA can prevent iron‐induced retinal degeneration by inhibiting oxidation of DHA. D‐DHA may be a viable therapeutic for retinal pathogenesis involving oxidative stress and lipid peroxidation.

## 4 MATERIALS AND METHODS

### 4.1 D‐DHA synthesis

D‐DHA was synthesized as previously described (Smarun et al., 2017). Catalytic exchange results in an assortment of D‐DHA isotopologues from D6‐D12, centered at D10 which is typically 30–40% of the total *bis*‐allylic isotopologues. At least 90% of D‐DHA is reinforced with two Ds at all *bis*‐allylic carbons, and the remaining 10% are reinforced with at least one D at each of the *bis*‐allylic positions.

### 4.2 Ocular D‐DHA accretion and elimination

Eleven‐week‐old C57BL/6J mice were purchased from Jackson Laboratory (Bar Harbor, ME) and housed in 20–25 lux light conditions under 12 hr day/night cycle in the Dean McGee Eye Institute Animal Research Facility at the University of Oklahoma Health Sciences Center, Oklahoma City, OK. One week after acclimatization to the *vivarium* with *ad libitum* access to laboratory rodent chow and water the animals were assigned to experimental groups. To determine ocular D‐DHA accretion, the mice were switched from the laboratory rodent diet to the experimental D‐DHA supplemented diet containing 0.5% D‐DHA plus 6.5% high oleic soybean oil (w/w) in AIN93G (Reeves et al., 1993) (Research Diet, Inc.). The diets were vacuum packaged and stored at −20°C. Food was replaced three times a week with fresh food *ad libitum* that is stored at 4°C after it is taken from −20°C. Based on previously estimated retina accretion kinetics from the literature (Moriguchi et al., 2001; Stinson et al., 1991), retina, optic nerve, and eyecups containing sclera and retinal pigment epithelium choroid (RPE‐choroid) were dissected from 6 mice (3 females and 3 males) at different time points during eleven weeks of D‐DHA feeding. The tissues were snap‐frozen in liquid nitrogen and stored at −80°C until fatty acid analyses. After eleven weeks (77 days) on D‐DHA diet, the animals were switched to the washout diet containing 0.5% DHA plus 6.5% high oleic soybean oil (w/w) in AIN93G and maintained on this diet until euthanasia at three different time points up to 74 days, when tissues were harvested for fatty acid analysis. All animal procedures were approved by the University of Oklahoma Health Sciences Center Institutional Animal Care and Use Committee.

### 4.3 Iron‐induced acute RPE atrophy

Adult male wild‐type C57BL/6J mice (Stock No.000664, Jackson Labs) were housed in standard conditions under cyclic light (12 hr:12 hr light‐dark cycle). Mice had *ad libitum* access to water and food. Beginning at 2mo of age, mice were placed on the AIN93G diet described above, supplemented with 0.25% D‐DHA or DHA (control diet) for 1, 2, 3, or 4 weeks prior to the intravitreal injection. The complete composition of the diets is shown in Table S1. Mice were given an intravitreal injection of 1 μl 0.5 mM ferric ammonium citrate diluted in 0.9% NaCl (saline) (MP Biomedicals LLC) or 1 μl of saline as control. Intravitreal injections were performed as previously described (Hadziahmetovic et al., 2011). Mice were continued on respective diet until their final evaluation. All housing and procedures were performed according to the NIH Guide for the Care and Use of Experimental Animals and approved by the University of Pennsylvania Animal Care and Use Committee.

### 4.4 Mass‐spectrometry

Three types of mass spectrometric analysis were performed to confirm repeatability of results. In the first, lipids were extracted from retinas by a modified Folch method (CHCl_3_/CH_3_OH, 2:1), derivatized to fatty acid methyl esters (FAME), and analyzed by high‐resolution capillary gas chromatography and specialized chemical ionization tandem mass spectrometry as discussed previously (Wang et al., 2020). Baseline resolved DHA and D‐DHA total ion signals were integrated, and the proportions of D‐DHA/total DHA were calculated.

The second type of mass spectrometric analysis was performed on samples of the control and experimental diets, as well as microdissected neural retina and RPE from animals on the experimental diet. Lipids were extracted and saponified as described previously (Axelsen & Murphy, 2010) and analyzed by ESI‐LC/MS on a 4000 QTrap (Sciex) operating in enhanced negative mode over an m/z range of 320–345 and a scan rate of 250 /sec. This analysis verified that the control diet contained DHA but no detectable D‐DHA, while the experimental diet contained an array of D‐DHA isotopologues but only trace amounts of natural DHA (Figure S1). Peaks corresponding to DHA with 8, 9, 10, 11, 12, and 13 deuterium substitutions were readily identified in the experimental diet, and in samples of neural retina and RPE. The relative distribution of DHA isotopologues in neural retina and RPE samples was indistinguishable from the relative distribution in the experimental D‐DHA supplemented diet.

The third type of mass spectrometric analysis was performed on microdissected neural retina and RPE from animals on the two diets using the same extraction and chromatographic procedures. However, ESI‐LC/MS analysis was performed in negative multiple reaction monitoring mode for transitions 327.2/283.2 (corresponding to 78.4% of ordinary DHA) and 337.2/293.2 (corresponding to 45.6% of the deuterium containing DHA). Results for D‐DHA are reported as a percentage of total DHA (i.e., D‐DHA + DHA).

### 4.5 *In vivo* imaging system

Mice were given general anesthesia and placed on a platform. Pupils were dilated with 1% tropicamide saline solution (Akorn, Inc.). Optical coherence tomography (OCT) imaging was performed for visualization of the retinal structure by using a Bioptigen Envisu (R2200, Bioptigen Inc.) coupled to broadband LED light source (T870‐HP, Superlum Diodes, Ltd.). Confocal scanning laser ophthalmoscopy (cSLO) (Spectralis HRA, Heidelberg Engineering) was used for visualization of retinal AF using BluePeak^TM^ or simply blue AF (488 nm excitation) and near‐infrared AF (787 nm excitation) imaging modes.

### 4.6 Electroretinography

Mice were dark adapted overnight and anesthetized with the same procedure. The electroretinograms were recorded with an Espion E3 system (Diagnosys LLC) with a ganzfeld Color Dome stimulator as previously described (Liu et al., 2021). All electroretinographies were performed at the same time of day.

### 4.7 Tissue preparation and immunofluorescence

Immunofluorescence on cryosections was conducted as described previously (Hadziahmetovic et al., 2011). Primary antibodies used: mouse anti‐CEP (1:200, a kind gift of John Crabb); rabbit anti‐rhodopsin (1:200; Abcam); rabbit anti‐L‐FT (1:1000, a kind gift of Maura Poli and Paolo Arosio, University of Brescia, Italy). Images were acquired with an epifluorescence microscope (Nikon 80i microscope, Nikon), and analyzed using NIS‐Elements (Nikon).

### 4.8 Plastic sections and Toluidine Blue Staining

Plastic sections (3 μm) were cut in the sagittal plane. The third eyelid was used for the orientation when embedding eyecups. Toluidine blue staining on plastic sections was used to evaluate retinal morphology as previously described (Hadziahmetovic et al., 2011).

### 4.9 RNA extraction and quantitative RT‐PCR

Neural retina tissues were isolated as previously described (Hadziahmetovic et al., 2011). Gene expression changes in the neural retina and purified RPE cells were evaluated. *Gapdh* was used as an endogenous control. TaqMan probes (ABI, Grand Island, NY, USA) were used as follows: *Rho* (Mm00520345), *Opn1mw* (Mm00433560), *Opn1sw* (Mm00432058), *Tfrc* (Mm00441941), *Slc7a11*(Mm00442530), *Gpx4* (Mm00515041), *GSS* (Mm00515065), *GSTm1* (Mm00833915), *Cat* (Mm00437992), *Sod1* (Mm01700393), *Hmox1* (Mm00516005), *Cd68* (Mm03047343), *IL*‐*1β* (Mm00434228), *IL*‐*6* (Mm00446190). The amount of target mRNA was compared among the groups of interest. All reactions were performed in technical (3 reactions per eye) triplicates and biological replicates (3–5 mice per genotype).

### 4.10 Statistical analysis

Statistical analyses were performed using GraphPad Prism 6.0 (San Diego, CA). One‐way analysis of variance (ANOVA) was performed, and post hoc analysis was employed using Tukey‐Kramer testing when differences were observed in ANOVA testing (*p* < 0.05). Mean ± SEM was calculated for each group.

## CONFLICTS OF INTEREST

MSS and KS are employed by Retrotope, Inc. JTB is a shareholder in and receives research support from Retrotope, Inc. MPA receives research support from Retrotope, Inc. The other authors declare that they have no commercial or financial relationships that could be construed as a potential conflict of interest.

## AUTHOR CONTRIBUTIONS

YL, BAB, and YS performed the experiments. YL, MSS, and JLD analyzed data. MPA and WB performed PK studies and tissue harvest. PHA, HGP, GJ, and JTB performed fatty acid analyses. JC contributed essential reagents. YL and JLD drafted the manuscript, and all authors provided critical review of the manuscript.

## Supporting information

Fig S1

Fig S2

Fig S3

Fig S4

Table S1

Supplementary Figures Legends

## Data Availability

The data that support the findings of this study are available from the corresponding author upon reasonable request.
