# Supplementary figures and images for: Deuterated docosahexaenoic acid protects against oxidative stress and geographic atrophy‐like retinal degeneration in a mouse model with iron overload
Source: Aging Cell. 2022 Mar 8;21(4):e13579. doi: 10.1111/acel.13579 (PMC9009113; doi:10.1111/acel.13579)

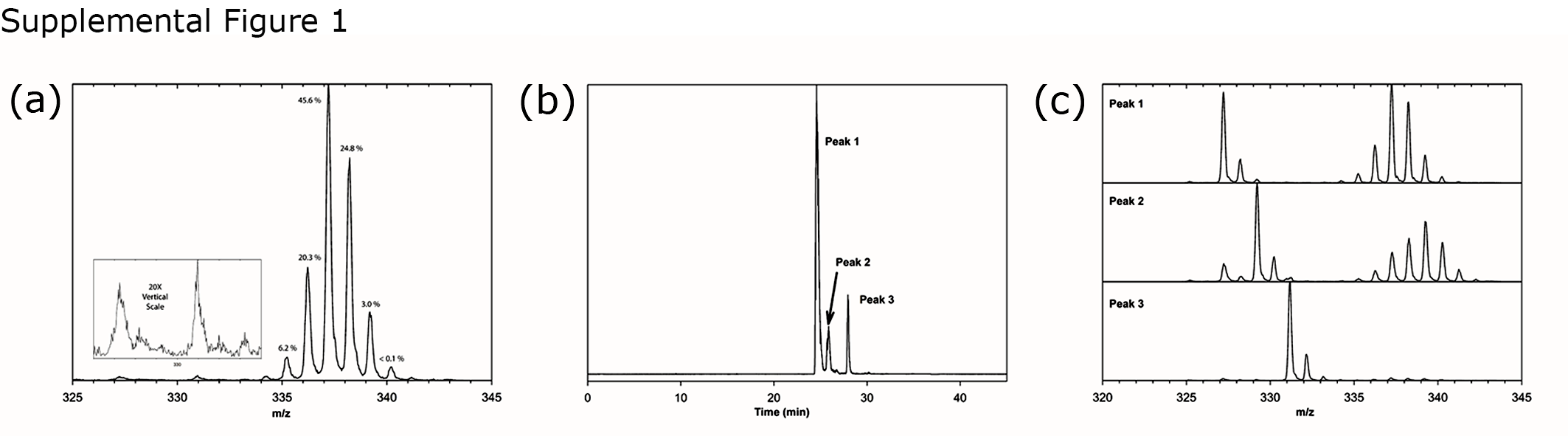

Supplement: Supplementary file 1 — Fig S1 [file ACEL-21-e13579-s002.png]

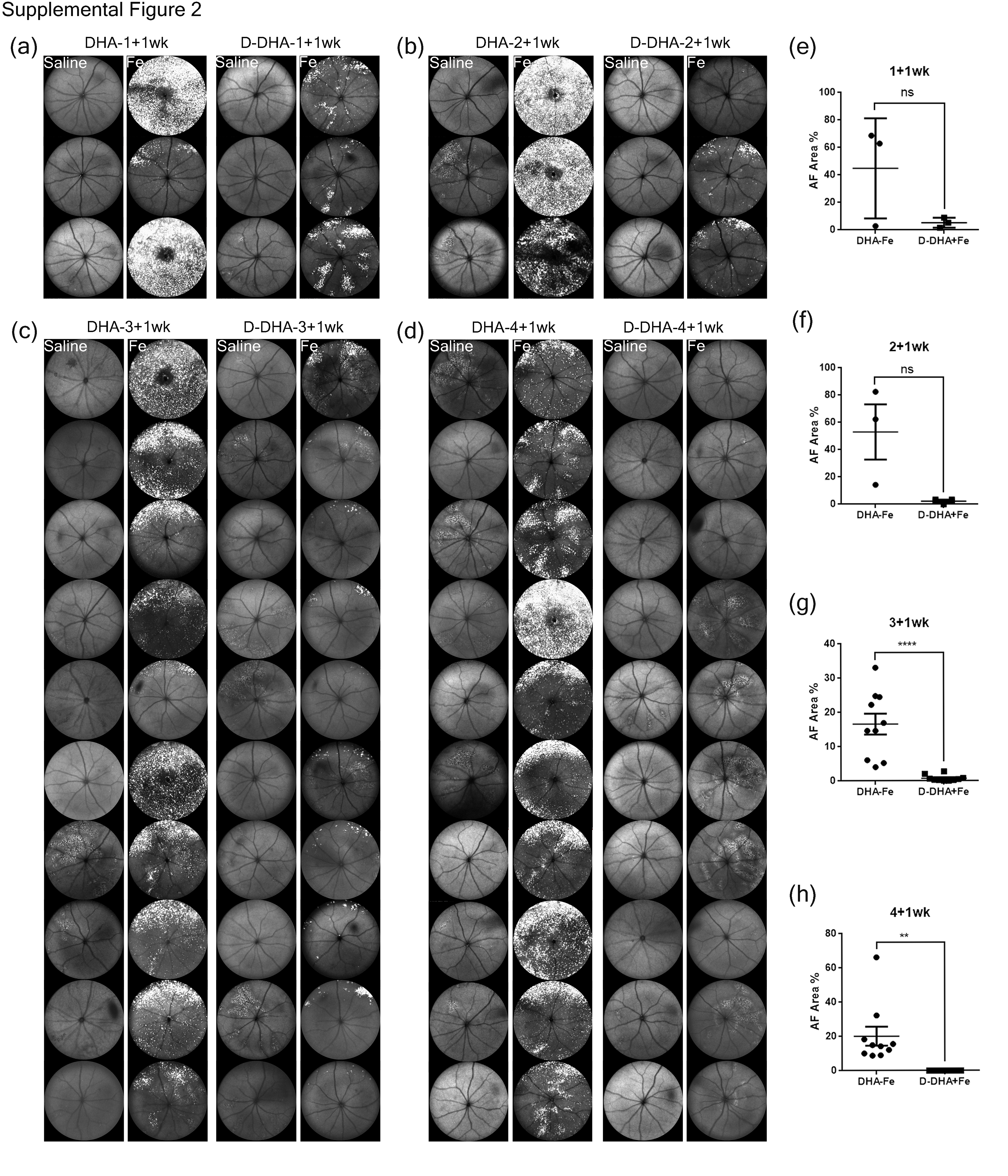

Supplement: Supplementary file 2 — Fig S2 [file ACEL-21-e13579-s004.png]

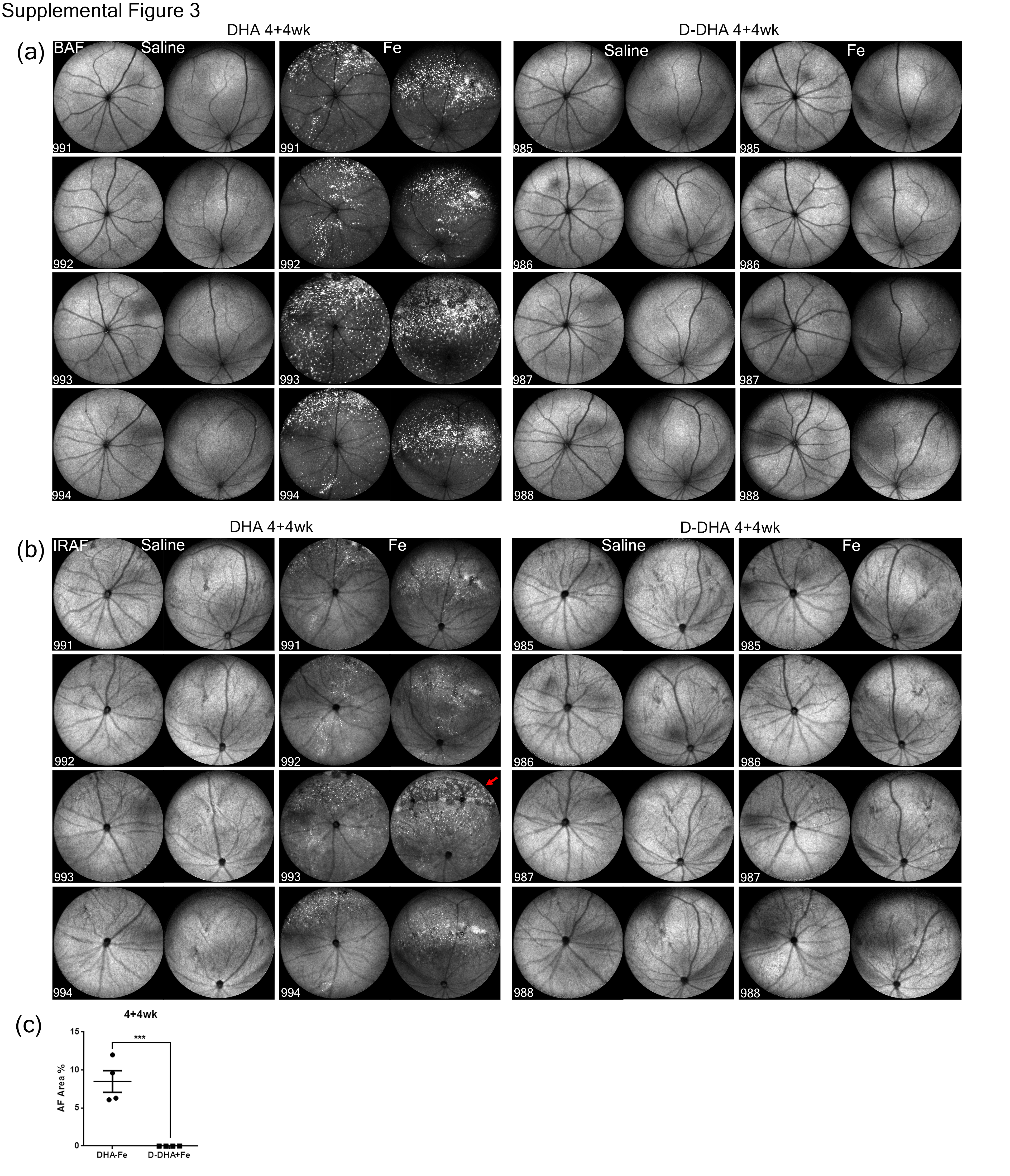

Supplement: Supplementary file 3 — Fig S3 [file ACEL-21-e13579-s001.png]

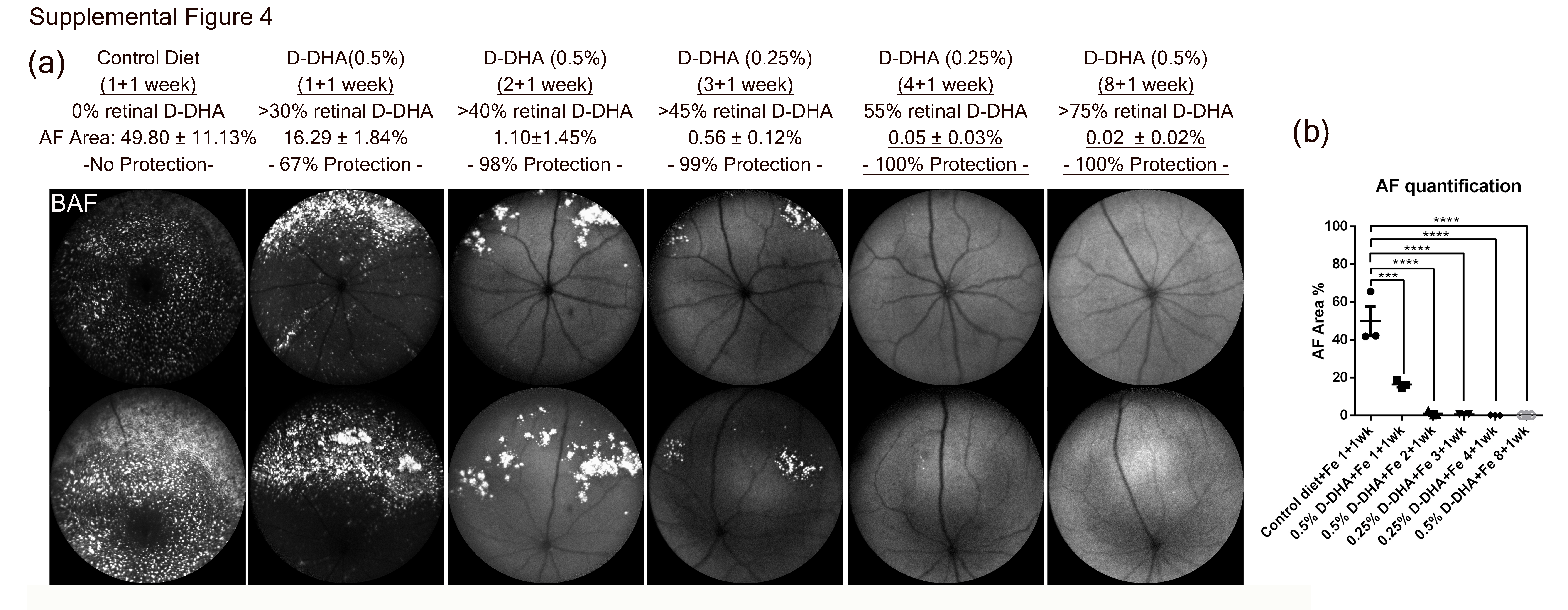

Supplement: Supplementary file 4 — Fig S4 [file ACEL-21-e13579-s005.png]

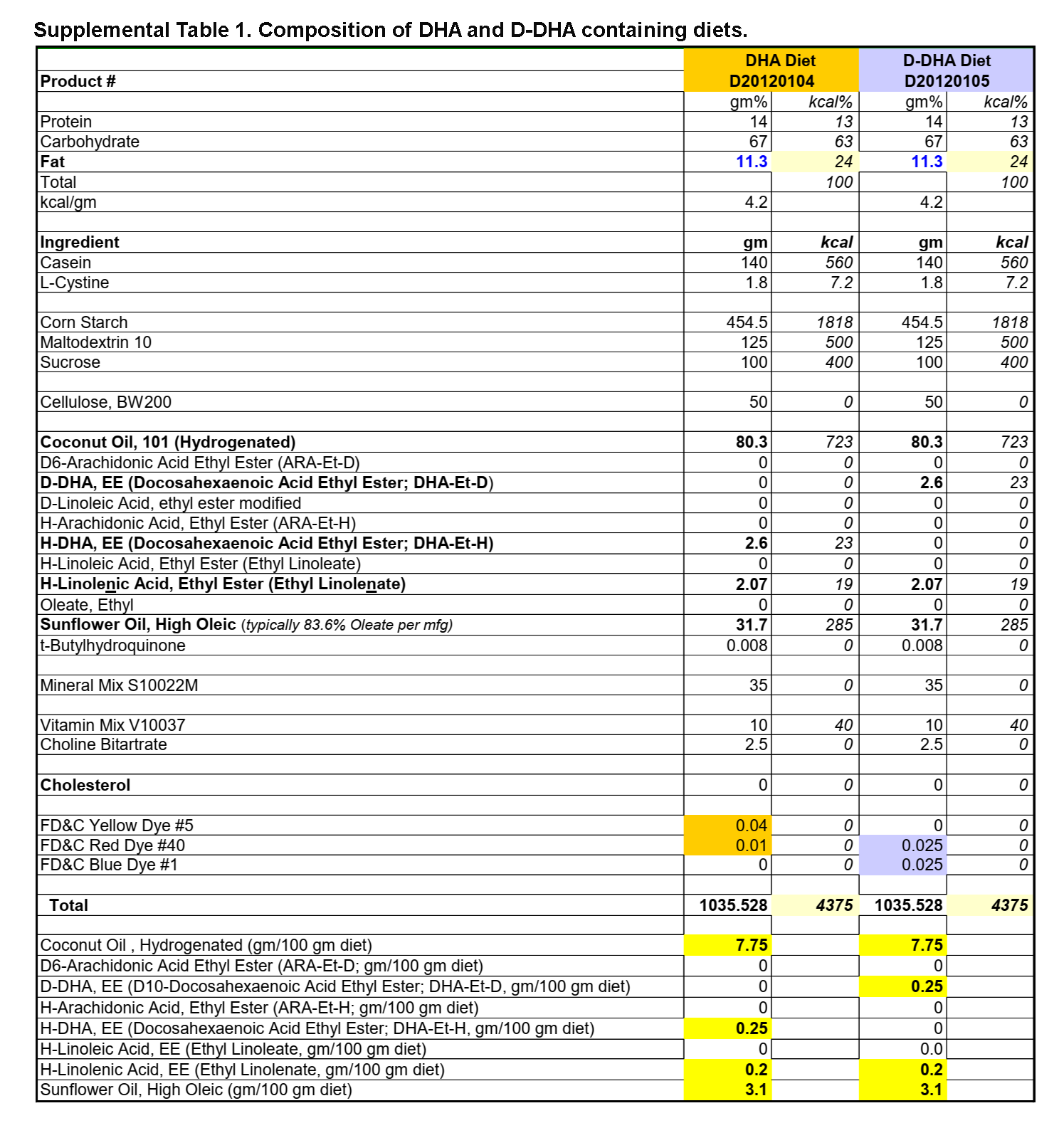

Supplement: Supplementary file 5 — Table S1 [file ACEL-21-e13579-s003.png]
