# Supplementary material for: Deuterated docosahexaenoic acid protects against oxidative stress and geographic atrophy‐like retinal degeneration in a mouse model with iron overload
Source: Aging Cell. 2022 Mar 8;21(4):e13579. doi: 10.1111/acel.13579 (PMC9009113; doi:10.1111/acel.13579)
Supplement: Supplementary file 6 — Supplementary Figures Legends [file ACEL-21-e13579-s006.docx]

**Supplemental Figure 1.** **LC/MS analysis of lipids extracted from D-DHA-containing diet.**

Free fatty acid mixtures resulting from sample extraction and saponification were dissolved in ethanol and injected in 5μl volumes onto an Agilent XDB-C18 liquid chromatography column (1 mm x 150 mm) through which running solvents were pumped at 100 µl/min. Solvent A was 70% CH_3_CN (v/v) and 0.1% formic acid (m/v). Solvent B was 0.1% formic acid (m/v) in neat CH_3_CN. The initial composition of the running solvent was 70% B for 5 min, increasing to 100% B between 5 and 30 min. DHA eluted at 24.7 min. The column effluent was alkalinized with 150 mM NH_4_OH before ESI-MS analysis on a 4000 QTrap (Sciex) operating in enhanced negative mode over an m/z range of 320 – 345 and a scan rate of 250 /sec.

These procedures verified that laboratory rodent diet contained DHA but no detectable D-DHA (not shown), while the experimental D-DHA supplemented diet contained only trace amounts of ordinary DHA (a). A ^13^C correction applied to the DHA signals verified that the peak at *m/z* 327.2 represented 78.4 % of the DHA and ^13^C-containing isotopologues. Peaks corresponding to DHA with 8, 9, 10, 11, 12, and 13 deuterium substitutions were readily identified in the experimental diet, and in samples of neural retina and RPE. The relative distribution of DHA isotopologues in neural retina and RPE samples was indistinguishable from the relative distribution in the experimental D-DHA supplemented diet. After ^13^C corrections were applied to the integrated peaks, it was determined that the area of the peak centered at 337.2 (corresponding to D_10_-DHA) comprised 45.6 % of the area of all deuterium-containing DHA isotopologue peaks.

Fatty acids extracted from the neural retina and RPE eluted as 3 peaks (b). The TIC shown was derived from enhanced negative mode scans from *m/z* 320-345 at 250 *m/z*/min. Mass spectra for the three labeled peaks (c). Peak 1 shows DHA at 327.2, a ^13^C-containing isotopologue at 328.2, and isotopologues containing 8, 9, 10, 11, and 12 deuterium substitutions at corresponding *m/z* values. Relative peak areas were indistinguishable from the relative peak areas observed in the experimental diet. Peak 2 shows some DHA (a tail from peak 1), 329.2/330.2 peaks indicating DPA, and a set of peaks suggesting that they represented D_8_-DPA, D_9_-DPA, D_10_-DPA, D_11_-DPA, and D_12_-DPA. Because these species were not present in the chow, they appear to represent D-DHA species that have been converted to D-DPA species. The relative peak areas of D_8_-DPA and D_9_-DPA were slightly greater than the relative peak areas of D_8_-DHA and D_9_-DHA, possibly reflecting greater likelihood of reduction to the corresponding DPA species when the degree of deuterium substitution is lower. Peak 3 eluting at 27.9 min most likely represents docosatetraenoic acid (DTA) of the n-6 series, and no deuterium-substituted isotopologues were observed.

**Supplemental Figure 2: D-DHA showed a dose-dependent protection effect against iron induced retinal AF.**

Beginning at 2 months age, mice were fed with D-DHA or DHA for 1 week, 2 weeks, 3 weeks, and 4 weeks before receiving an intravitreal injection of iron in one eye and control normal saline in the other. At 1 week after iron versus saline injection, BAF cSLO images were acquired from multiple mice fed with D-DHA or DHA for 1 week (1+1wk) (a), 2 weeks (2+1wk) (b), 3 weeks (3+1wk) (c), and 4 weeks (4+1wk) (d) prior to injections. BAF cSLO pairs of images from the same mouse were presented in the same row, images from different mice with the same treatment (iron or saline) were presented in the same column. Graphs show retinal AF area in BAF cSLO images (e-h). ( ** *P* < 0.01, **** *P* <0.0001).

**Supplemental Figure 3. Long term protective effect of D-DHA against chronic geographic atrophy development.**

Mice were fed with D-DHA or DHA for 4 weeks before receiving intravitreal injection of iron in one eye and control normal saline in the other. BAF cSLO images (a) and IRAF cSLO images (b) were acquired at 4 weeks after injections. Pairs of BAF/IRAF images from the same mouse were presented in the same row, images from different mice with the same treatment (iron or saline) were presented in the same column. Graph shows retinal AF area in BAF cSLO images (c). (*** *P* <0.001).

**Supplemental Figure 4. Dose-response of D-DHA protection against iron induced retinal damage.**

Representative cSLO BAF images from animals fed with control diet (left column) and D-DHA diets for increasing periods of time before intravitreal injection of FAC. Images were acquired one week after iron injection. Retinal D-DHA levels at 4+1 weeks as measured (see Table 1); levels at other time points were extrapolated assuming a 1^st^ order uptake kinetics as shown in Fig. 2b. Appearance of autofluorescent spots served as quantitative measure of damage or protection by D-DHA Protection effect refers to the reduction (%) of AF area quantified by ImageJ software, N=3-5/group. Graph shows retinal AF area in BAF cSLO images (b). (*** *P* <0.001, **** *P* <0.0001).
